# Coherent control of light-matter interactions in polarization standing waves

**DOI:** 10.1038/srep31141

**Published:** 2016-08-12

**Authors:** Xu Fang, Kevin F. MacDonald, Eric Plum, Nikolay I. Zheludev

**Affiliations:** 1 Optoelectronics Research Centre & Centre for Photonic Metamaterials, University of Southampton, Southampton, SO17 1BJ, UK; 2 Centre for Disruptive Photonic Technologies, School of Physical and Mathematical Sciences & The Photonics Institute, Nanyang Technological University, 637371, Singapore

## Abstract

We experimentally demonstrate that standing waves formed by two coherent counter-propagating light waves can take a variety of forms, offering new approaches to the interrogation and control of polarization-sensitive light-matter interactions in ultrathin (subwavelength thickness) media. In contrast to familiar energy standing waves, polarization standing waves have constant electric and magnetic energy densities and a periodically varying polarization state along the wave axis. counterintuitively, anisotropic ultrathin (meta)materials can be made sensitive or insensitive to such polarization variations by adjusting their azimuthal angle.

Metamaterials and metasurfaces, artificial media with prescribed optical properties that are often unavailable or indeed inconceivable in naturally-occurring materials, can now be engineered to requirement from the atomic scale up^1^,^2^. A variety of schemes for dynamically controlling, switching and tuning these properties have been demonstrated, including approaches based upon the hybridization of resonant nanostructures with active (e.g. nonlinear and phase-change) media^3^,^4^,^5^,^6^,^7^ and on mechanically-, electrically- or even optically-induced changes in the geometry or spatial arrangement of constituent metamolecules^8^,^9^,^10^,^11^,^12^,^13^. It has also been shown recently that the manifestation of optical properties in planar metamaterials can be ‘coherently controlled’: The interference of coherent light beams on ultra-thin nanostructured metasurfaces, much thinner than the wavelength of light, can for example selectively render the medium almost perfectly transparent or facilitate near-perfect optical absorption at wavelengths selected by design, thereby enabling multi-THz bandwidth light-by-light modulation at arbitrarily low intensities^14^,^15^,^16^,^17^. In fact, all kinds of light-matter interaction in films of sub-wavelength thickness, including birefringence, dichroism, and nonlinear effects, can be similarly modulated by manipulating the relative spatial and/or temporal intensity and phase distributions of incident light beams^18^,^19^,^20^,^21^,^22^; the concept can be harnessed for data and image processing applications^23^,^24^, and in spectroscopy to disentangle electric and magnetic resonances^25^. In all of these cases, coherent control is achieved by manipulating the precise location of the metasurface within the conventional standing wave pattern of electric/magnetic field nodes and anti-nodes formed by a pair of collinearly-polarized incident beams.

Here, we demonstrate that standing waves formed by two coherent counter-propagating waves of equal intensity can take different forms (Fig. 1). In the first category, which we call Energy Standing Waves (ESWs), the local electric and magnetic energy densities change along the wave axis, while the total energy density remains constant as nodes of electric field correspond to anti-nodes of magnetic field and vice versa. The local polarization state of ESWs is invariant along the wave axis. For instance, the standing wave formed by a pair of counter-propagating collinearly polarized waves maintains the same linear polarization at all points along the wave (see Fig. 1a). Similarly, a standing wave formed by a pair of counter-propagating circularly polarized waves of opposite handedness maintains a circular polarization: At all points in space the end of electric field vector traces a circle following the shared rotation direction of the two incident waves (see Fig. 1b).

In the second category of standing wave, which we will call Polarization Standing Waves (PSWs), the local polarization state oscillates along the wave axis while the electric, magnetic and total energy densities are invariant. For instance, in the standing wave that is formed by counter-propagating orthogonally linearly polarized waves, the local polarization state is an axial position-dependent ellipse. The degree of ellipticity oscillates, and its azimuth flips alternately between orientations at ±45° to the incident polarizations, along the wave axis with a period of λ/2 (λ being the wavelength; see Fig. 1c). Similarly, in a standing wave formed by a pair of counter-propagating circularly polarized waves of the same handedness (as in a cavity formed by ‘handedness-preserving’ mirrors)^26^, the polarization state is linear with an azimuthal orientation that oscillates along the wave axis with a period λ/2 (see Fig. 1d).

Using an anisotropic plasmonic metamaterial film of subwavelength thickness, we have experimentally studied light absorption in the energy and polarization standing waves formed by counter-propagating linearly polarized input beams. Absorption in the ESW oscillates with the sample’s axial position in the wave. Counterintuitively however, it is seen that the absorption of the anisotropic film, which normally depends strongly on polarization, may or may not be sensitive to the polarization changes in the PSW depending on the orientation of the sample.

The behaviour of a subwavelength thickness (<<λ) metamaterial film in such standing waves can be understood in terms of a scattering matrix. If the film is located in the *xy* plane and incident light propagates in the +*z* direction, the scattering matrix can be written as


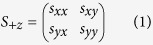


where *s*_*αβ*_ is a complex number describing the *α*-polarized scattered field induced by a *β*-polarized incident field (*α*, *β* = *x*, *y*). Since a planar medium illuminated at normal incidence senses only the local electric field^27^, the corresponding scattering matrix *S*_−*z*_ for the opposing direction of incident light propagation is the same: *S*_−*z*_ = *S*_+*z*_. Moreover, reciprocity requires^28^ that the matrices for opposing propagation directions transpose: *S*_−*z*_ = (*S*_+*z*_)^*T*^. This combination of properties implies that the off-diagonal terms must be equal, i.e. *S*_*yx*_ = *S*_*xy*_.

We first consider a sample in the ESW formed by two counter-propagating waves co-polarized in the *x* direction. The electric field of the travelling waves is 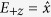 and 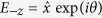, where 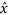 is the unit vector in the *x* direction, and *θ* is the phase difference between the two waves at the sample position. A lossy thin film placed in the standing wave will absorb radiation from both propagating waves. The rate of dissipation will be controlled by the position of a thin film in the standing wave, which is to say by the phase difference *θ.* We define absorption *A* as the fraction of energy dissipated by the film from both waves:


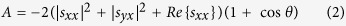


From here one can see that the absorption of a lossy sample always depends on *θ*, i.e. on the sample’s position in the ESW.

In contrast, absorption in a PSW formed by orthogonally polarized waves with incident fields 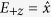 and 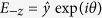 is


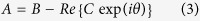


where *B* and *C* are


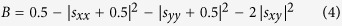



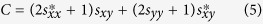


From Eq. (3), it follows that an anisotropic sample will generally be sensitive to its position in a PSW. However, if *C* = 0, absorption *A* is independent of *θ*, and thus of the position in the standing wave. This condition can be satisfied if *s*_*xy*_ = 0 (i.e. in the absence of linear polarization conversion).

One may then ask under what circumstances the off-diagonal components *s*_*xy*_ = *s*_*yx*_ of the scattering matrix of the film can be zero: For an anisotropic film, the scattering matrix generally has non-zero and complex components. The real and imaginary parts of the matrix can each be diagonalized at selected azimuthal rotation positions about the *z*-axis. The phenomenon of asymmetric transmission^27^, which has recently drawn considerable attention. Emerges in “planar chiral” patterns that do not have a line of symmetry in the sample plane, for which these azimuthal angles are different. Here, we consider a pattern that does have a line of symmetry and as such the real and imaginary components of its scattering matrix can be diagonalized at the same azimuthal angle. Practically, this means that the film has a pair of orthogonal linear polarization eigenstates. If the sample is oriented in such a way that the two incident waves forming a PSW are aligned with these eigenstates, then *s*_*xy*_ = *s*_*yx*_ = 0 and absorption *A* will be independent of *θ* and thereby of position in the PSW.

We performed a series of experiments on coherent light absorption in the energy and polarization standing waves formed by linearly polarized travelling waves (as shown in Fig. 1a,c). We studied the axial position-dependent absorption of the mirror-symmetric, anisotropic, planar plasmonic metasurface illustrated in Fig. 2, comprising a 30 nm thick gold film supported on a 30 nm thick Si_3_N_4_ membrane, patterned with a periodic array of symmetric L-slots (with equal arm lengths of 300 nm) – a geometry selected for the distinct dependence of its optical response to the polarization state of incident light (see Fig. 3 below). Experimental samples prepared by focused ion beam (FIB) milling consisted of 75 × 75 unit cells, each with dimensions of 500 nm × 500 nm. The orthogonal linear polarization eigenstates of this pattern are aligned parallel and perpendicular to the mirror symmetry axis of the L-slots. Detailed structural dimensions, as used in FIB pattern design and in 3D computational modeling of the metamaterial (using the COMSOL Multiphysics finite element solver) are presented in Fig. 2(b). These simulations assume normally incident narrowband plane wave illumination and, by virtue of periodic boundary conditions, an array of infinite extent in the *xy* plane. They derive the permittivity of gold from a Drude-Lorentz model^29^, using a damping term three times that of bulk gold to account for thin film surface roughness^30^,^31^,^32^. For simplicity and symmetry, they exclude the Si_3_N_4_ substrate – a measure of minor consequence to computational results and their correlation with experimental measurements, as will be seen below.

Figure 3a,b show the measured normal-incidence travelling-wave (single incident beam) reflection, transmission, and absorption spectra of the metamaterial for its linear polarization eigenstates; Fig. 3c shows spectra for incident light polarized parallel to the arms of the L-slots; Fig. 3d–f show corresponding numerically simulated spectra. The correlation is excellent, save for a small systematic blue-shift of the simulated spectra resulting from the model’s omission of the silicon nitride membrane, thereby confirming that the sample’s optical response is dominated by that of the 30 nm (*λ*/27) thick gold layer.

We subsequently investigated the absorption of the film in both energy and polarization standing waves (Fig. 4) - evaluated as the difference between the total power of the two counter-propagating incident beams and the total power of the output (reflected and transmitted) beams leaving the sample in either direction. Experiments were performed with a mode-locked Ti:sapphire laser, at a wavelength of 880 nm for which the sample’s optical properties are strongly anisotropic (as illustrated by Fig. 3a,b), using the instrumental configuration described in ref. [15] only with a half-wave plate inserted in one of the input beam paths to enable switching between ESW and PSW illumination modes, as illustrated in Supplementary Figure S2. In summary, the laser output is split to form two beams that are focused at normal incidence from opposing directions onto the metamaterial sample. A piezoelectric delay line in one of the beam paths is employed to control the phase difference *θ* between the two beams at the sample and thereby to set/tune the position of the sample within the standing wave light field. The two output beams (transmitted and reflected from both sides of the sample) are directed to a pair of identical photodiodes.

Absorption was first measured in the ESW formed by waves co-polarized perpendicular to the mirror symmetry axis of the sample, i.e. in the polarization eigenstate for which travelling wave absorption is maximized at 880 nm. In keeping with the behaviour reported in ref. [15] (for an asymmetric split ring resonator array metasurface), absorption is seen to oscillate between coherently-suppressed and -enhanced levels at the electric field nodes and anti-nodes of the ESW respectively as a function of the time delay between (i.e. mutual phase of) the pulses at the sample position, within an envelope defined by their temporal overlap. Figure 4a shows data from the zero-delay position (identified as that for which the absorption modulation amplitude is maximized) overlaid with a dependence derived from numerical modelling (based upon continuous as opposed to pulsed illumination, though the two can be assumed equivalent at zero pulse delay^15^). The correlation is excellent, with experimental deviations from the expected coherent absorption limits of zero and twice the travelling wave level being attributed primarily to sample imperfections including surface roughness (the resultant incoherent scattering is assumed within travelling wave absorption levels derived from specular reflection and transmission measurements but makes no contribution to coherent absorption^15^).

When the sample is rotated 45° such that the incident beam, and therefore ESW, polarization directions are aligned with one arm of the symmetric L-slots, there is no qualitative change in the pattern of coherent absorption modulation (see Fig. 4b): At zero delay it oscillates as above between a coherently-suppressed level (nominally zero) at the ESW electric field nodes and a coherently-enhanced level at the anti-nodes, now equal to twice the travelling wave absorption level for 880 nm light polarized parallel to the arms of the symmetric L-slots (see Fig. 3c). This behaviour is replicated for any sample orientation in an ESW - the maximum level of coherent absorption being the only parameter that changes with orientation.

In contrast, in the PSW formed by counter-propagating linearly polarized waves, the phenomenology of coherent absorption modulation is strongly dependent on the anisotropic sample’s orientation: On one hand, when the orthogonally polarized incident beams are the sample’s polarization eigenstates (see Fig. 4c), neither is subject to polarization conversion on scattering from the sample, and both therefore propagate (ideally) as if the other were not present. As such there should be no coherent absorption modulation (in Eqs 3, 4, 5, *s*_*xy*_ = 0 therefore *C* = 0) - the level is expected to be constant and equal to the mean level of travelling wave absorption for the two incident beams, regardless of their mutual phase. The small deviations about this level seen experimentally are a consequence of sample and systematic experimental imperfections (further discussed below). On the other hand, when the incident beams are polarized parallel to the two L-slot arms (see Fig. 4d), they are individually subject to polarization conversion on scattering (*s*_*xy*_ ≠ 0) and can thus mutually interact. Coherent absorption modulation is observed once more within the envelope of the temporal overlap between counter-propagating pulses, but now as a result of the oscillation of the *local* polarization state. The local linear states within the PSW are aligned (at 45° to the incident states) with the metamaterial’s polarization eigenstates and these in principle set the upper and lower limits on the level of coherent absorption; the local left- and right-circular states present identical intermediate levels of absorption, as one would expect in the absence of planar chirality. The fact that the level of coherent absorption reaches the travelling wave limits confirms that the metamaterial is indeed responsive only to the local polarization state regardless to whether that state exists as part of a standing or travelling wave.

The essential absence of modulation in Fig. 4c can thus be understood from the perspective that the local linear polarization states within the PSW are nominally degenerate for this sample orientation - they are aligned with the two arms of the symmetric L-slots and in principle experience identical levels of absorption. Every intermediate elliptical and circular local polarization state can be described as a linear superposition of these two states and must therefore also be subject to the same level of absorption. The small periodic deviation from this ideal observed experimentally is ascribed to small inaccuracies in the sample’s azimuthal orientation and slight systematic (fabrication process-related) asymmetry in the metamaterial L-slot pattern, which gives rise to a slight difference between levels of absorption for polarizations parallel to the two arms (see Supplementary Figure S1).

In summary, we have shown that the interactions of an ultrathin film with different types of standing wave can vary radically. In particular, we have identified an unusual regime in which light absorption in an anisotropic sample is independent of its axial position in a polarization standing wave. This independence is related to the fact that the electric energy density in such wave is homogeneous along the wave axis. This behaviour stands in sharp contrast to absorption of a lossy ultrathin film in a conventional energy standing wave, which invariably depends on the position of the film with respect to standing wave nodes and anti-nodes.

The polarization standing wave employed in this experimental study is locally 2D-chiral but 3D-achiral^33^. In general, the properties of a lossy ultrathin medium in this PSW will depend on both the sample’s azimuthal orientation and the mutual phase of the incident beams if it is anisotropic. If the medium also exhibits 2D-chirality, the amplitude of modulation with phase will be non-zero for all azimuth angles (because the incident linear polarization states cannot be aligned with the co-rotating elliptical eigenstates^27^ of such structures). Manifestations of 3D chirality are entirely precluded in this PSW configuration because the handedness of the local circular and elliptical states within the PSW (see Fig. 1c) cannot be defined. The behaviour of planar media in other symmetry classes is analysed in the Supplementary Information.

Polarization standing waves add a new dimension to the coherent control paradigm for light-matter interactions in ultrathin media. They offer an additional degree of freedom, alongside selectivity based on the magnetic/electric nature of incident fields^25^, in coherent-illumination spectroscopy (important differences between travelling and energy standing wave illumination modes having been previously noted in cavity-based techniques^34^,^35^), and the concept may be harnessed in coherent communications networks where data is encoded not only in the amplitude^36^, but also the phase and polarization of signals.

## Additional Information

**How to cite this article**: Fang, X. *et al*. Coherent control of light-matter interactions in polarization standing waves. *Sci. Rep.* **6**, 31141; doi: 10.1038/srep31141 (2016).

## Supplementary Material

Supplementary Information

## Figures and Tables

**Figure 1:**
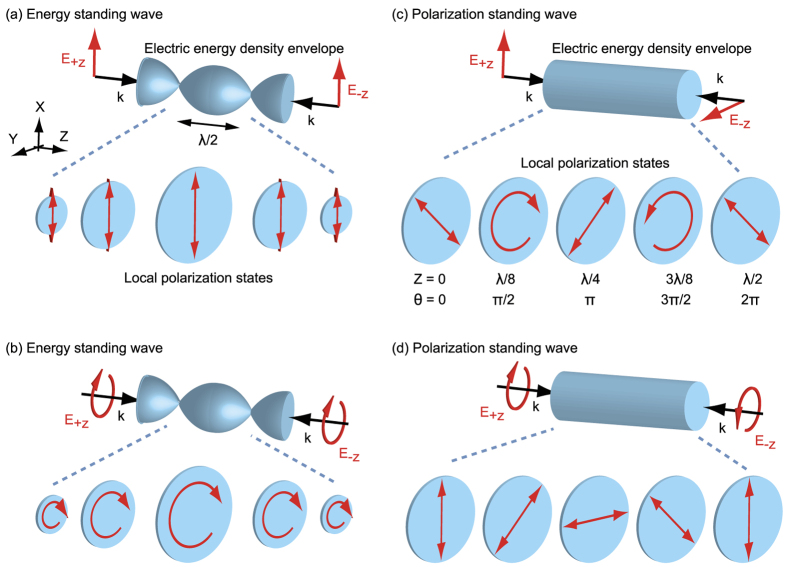
Energy and polarization standing wave schematics showing in each case the envelope of electric energy density and the evolution of local polarization state: (**a**,**b**) Energy standing waves formed by two counterpropagating (**a**) collinearly polarized waves and (**b**) circularly polarized waves of opposite handedness. (**c**,**d**) Polarization standing waves formed by two counter-propagating (**c**) orthogonally linearly polarized waves and (**d**) circularly polarized waves of the same handedness.

**Figure 2:**
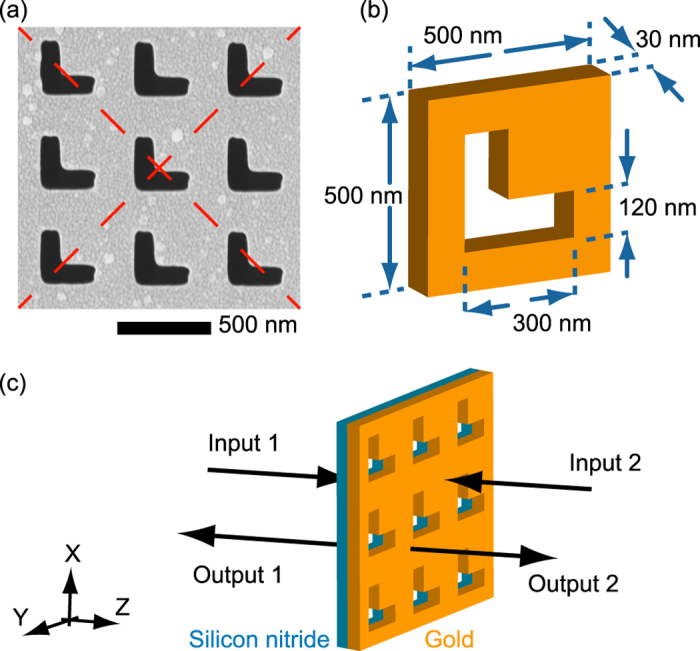
(**a**) Scanning electron microscope image of a section of the experimental planar metamaterial sample, comprising 30 nm Au on a 30 nm Si_3_N_4_ membrane [imaged from the Au side]. The dashed red lines indicate the linear polarization eigenstate directions. (**b**) Dimensional schematic of the symmetric L-slot metamaterial unit cell design [excluding the membrane substrate as in numerical simulations]. (**c**) Coherent, i.e. standing wave, illumination configuration schematic.

**Figure 3:**
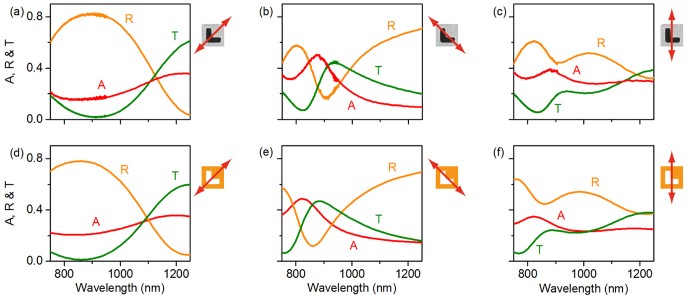
Optical properties of the symmetric L-slot metamaterial under travelling wave illumination: (**a**–**c**) Experimentally measured and (**d**–**f**) corresponding numerically simulated normal incidence reflection *R*, transmission *T*, and absorption *A* [=*1* − (*R* + *T*)] spectra for incident light polarized [as indicated by the inset schematics] parallel (**a**,**d**) and perpendicular (**b**,**e**) to the structure’s mirror symmetry axis – its polarization eigenstates, and parallel to the arms of the L-slots (**c**,**f**). [Experimental spectra were evaluated using a microspectrophotometer [CRAIC QDI2010; absolute measurement uncertainty ±<0.03] with light incident on the gold side of the sample; Data in panel (c) is averaged over the two equivalent orientations – see Supplementary Figure S1.]

**Figure 4:**
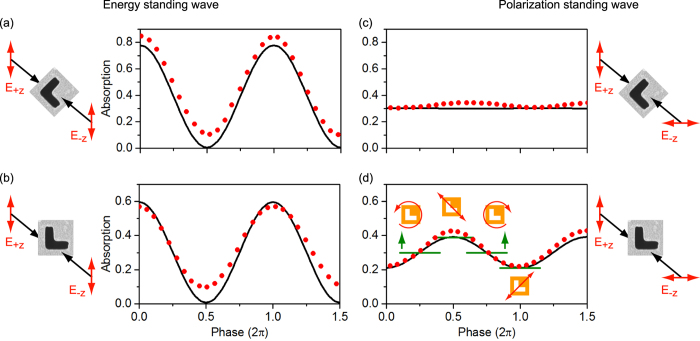
Coherent absorption in (**a**,**b**) energy and (**c**,**d**) polarization standing waves. In each case experimental data (red dots; characteristic uncertainty in absorption ±0.005) for one and a half cycles of the standing wave [at around zero temporal delay between incident counter-propagating pulses] are overlaid with numerically simulated traces (black lines). Schematics to the left and right illustrate the mutual orientation of the sample and incident beam polarization directions. Panel (d) is annotated with markers (green lines with schematic tags) denoting the levels of travelling wave absorption for polarizations matching the local linear and circular polarization states found within the polarization standing wave. [In the absence of meaningful reference points in null-response measured and simulated data of panel (c), the zero phase position is set arbitrarily.]
