# Supplementary material for: Coherent control of light-matter interactions in polarization standing waves
Source: Sci Rep. 2016 Aug 12;6:31141. doi: 10.1038/srep31141 (PMC4981885; doi:10.1038/srep31141)
Supplement: Supplementary Information [file srep31141-s1.pdf]

Coherent control of light-matter interactions in polarization standing waves: Supplementary Information

Xu Fang,[1] Kevin F. MacDonald,[1,*] Eric Plum,[1] and Nikolay I. Zheludev[1,2]

[1] Optoelectronics Research Centre & Centre for Photonic Metamaterials, University of Southampton, Southampton, SO17 1BJ, UK

[2] Centre for Disruptive Photonic Technologies, School of Physical and Mathematical Sciences & The Photonics Institute, Nanyang Technological University, 637371, Singapore

Experimentally measured metasurface optical properties for incident light polarizations parallel to the nominally identical arms of the L-slot design

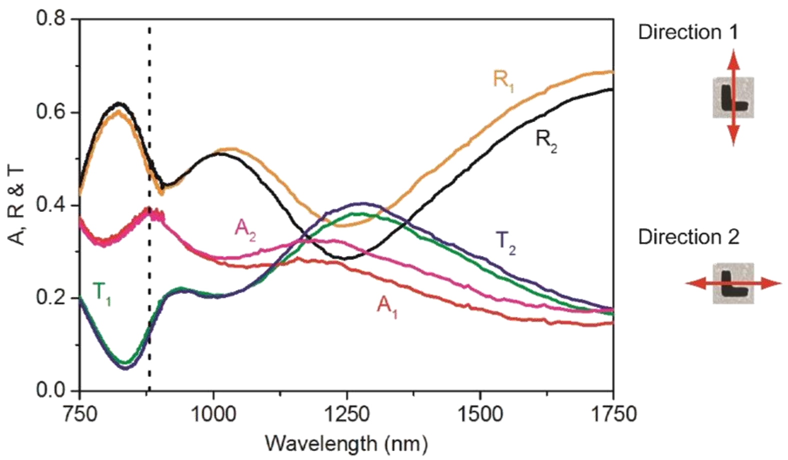

Figure S1. Measured normal incidence reflection *R*, transmission *T*, and absorption *A* spectra of the experimental metamaterial sample for the nominally degenerate incident light polarizations parallel to the two arms of the L-slots [denoted by subscripts 1 and 2]. The differences observed [minimal at the 880 nm wavelength employed in coherent illumination measurements, indicated by the dashed vertical line] are attributed to small but systematic manufacturing imperfections which break the ideal design symmetry of the structure.

Experimental configuration for measurements of coherent absorption in energy and polarization standing waves

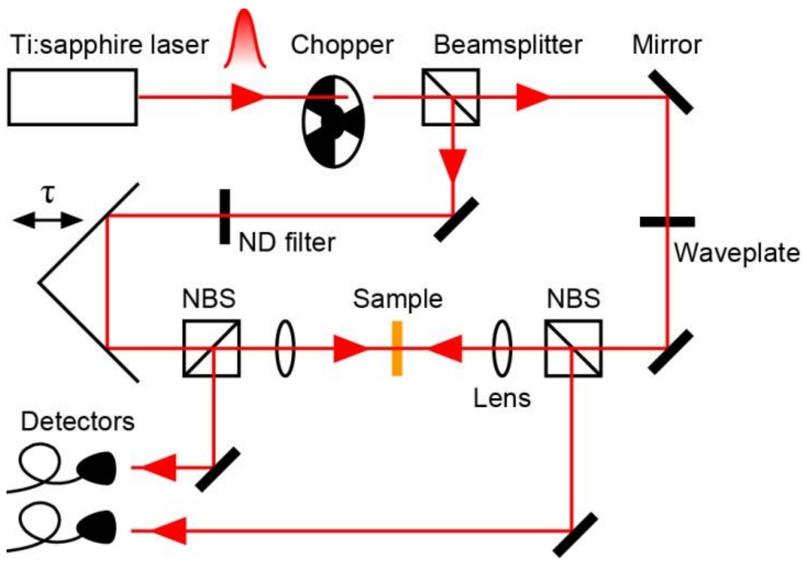

Figure S2. Schematic of the experimental arrangement for measurements of coherent absorption in ultrathin media. The beam from a mode-locked Ti:sapphire laser (130 fs pulse duration; 10-20 nm spectral FWHM) is modulated by a mechanical chopper and then split by a pellicle into two beams. A piezoelectric translation stage located in one beam path sets/tunes the relative time delay and thus the relative phase difference between pulses arriving at the sample from opposing sides. A half-wave plate in the other beam path controls the mutual orientation of the two beams' polarization to establish either an energy or a polarization standing wave at the sample. The two beams are focused at normal incidence from opposite sides, to a diameter ~10 µm, onto the sample using plano-convex lenses. Their average powers at the sample position are balanced using a variable neutral density filter (ND filter), and maintained below 1 mW per beam to exclude opto-thermal and nonlinear effects. The two output beams (transmitted and reflected from both sides of the sample) are directed via two non-polarizing beamsplitters (NBSs) to a pair of identical photodiodes, the signals from which were monitored using lock-in amplifiers referenced to the chopping frequency of 1.6 kHz.

Planar symmetries: Effect on scattering matrices and coherent absorption in polarization standing waves (PSWs)

An ultrathin sample can be categorized by its planar symmetry – whether it is or is not anisotropic and/or 2D-chiral. In the main body of this work we consider an anisotropic and 2D-achiral sample in an

anisotropic, 2D-chiral, and 3D-achiral PSW formed by counter-propagating orthogonally linearly polarized waves. Here, we use the scattering matrices $S_{\pm z}$ to elucidate the behaviour of lossy planar media with all possible planar symmetries in such a PSW.

As discussed, an ultrathin medium illuminated at normal incidence has scattering matrices of the general form

$$S_{+z} = S_{-z} = \begin{pmatrix} a & b \\ b & c \end{pmatrix} \qquad \text{(S1)}$$

where a, b and c are complex scattering parameters for linearly polarized waves. In what follows we show how a structure's planar symmetry can limit the number of free parameters in this set.

An ultrathin medium subjected to planar operations such as rotation around a point or reflection about a line in the xy-plane, is described by a new matrix $S'_{\pm z}$,

$$S'_{\pm z} = P S_{\pm z} P^{-1}$$

where P is the operation matrix. For example, counter-clockwise rotation around the origin by an angle φ is described by the rotation matrix P_{φ},

$$P_{\varphi} = \begin{pmatrix} \cos\varphi & -\sin\varphi \\ \sin\varphi & \cos\varphi \end{pmatrix}$$

Similarly, reflection in the x-axis is described by the reflection matrix P_x,

$$P_x = \begin{pmatrix} 1 & 0 \\ 0 & -1 \end{pmatrix}$$

Isotropic media

Homogeneous and (chiral or achiral) structured planar media with at least 3-fold rotational symmetry present isotropic scattering properties that do not depend on the azimuthal orientation. The relation

$$P_{\varphi} S_{\pm z} P_{\varphi}^{-1} = S_{\pm z}$$

must therefore hold true for any rotation angle φ. In principle, this condition is satisfied by a scattering matrix with identical diagonal elements, wherein one off-diagonal element is the negative of the other. However, as has already been established (see article main text), for a vanishingly thin medium the off-diagonal polarization conversion terms of the matrix must be identical (Eq. S1). So in this case they can only be zero:

$$S_{\pm z} = \begin{pmatrix} a & 0 \\ 0 & a \end{pmatrix}$$

Thus, isotropic planar media cannot exhibit coherent absorption modulation in a PSW formed by counter-propagating orthogonally linearly polarized waves – the material response will show no dependence on either the phase difference θ between incident waves or on the sample's azimuthal orientation.

Anisotropic and 2D-achiral media

The L-slot metasurface discussed in the main body of this work and employed for the experimental demonstration of coherent PSW absorption modulation is an example of an anisotropic and 2D-achiral structure. Patterns of this type always have a mirror symmetry axis.

If this line of mirror symmetry is oriented parallel to the x-axis, then the structure (and its scattering matrix) must be unaffected by reflection in the x-axis, i.e.

$$P_x S_{\pm z} P_x^{-1} = S_{\pm z}$$

In this and the orthogonal orientation, the off-diagonal (linear polarization conversion) terms of the scattering matrix for such samples thereby vanish, leaving

$$S_{\pm z} = \begin{pmatrix} a & 0 \\ 0 & c \end{pmatrix}$$

For intermediate azimuthal orientations of the sample the off-diagonal terms are non-zero.

Thus, in a PSW the coherent absorption of a lossy, anisotropic and 2D-achiral sample will be a function of the mutual phase θ of the incident beams, except when its mirror symmetry axis is aligned with one of the incident linear polarization states, in which case absorption is independent of θ (as seen in the reported experiments).

Anisotropic and 2D-chiral interfaces

Low symmetry anisotropic, planar chiral patterns of rotational order 1 or 2 generally place no constraints on the allowed scattering properties for normal incidence illumination, i.e. the parameters a, b and c in Eq. S1 remain mutually independent and may all be non-zero.

For such samples, coherent absorption in a PSW will be modulated with both the azimuthal orientation of the sample and the mutual phase θ of the incident beams. There will be no azimuthal orientation for which absorption is independent of θ.
